# A Biomimetic Microchannel Heat Sink for Enhanced Thermal Performance in Chip Cooling

**DOI:** 10.3390/biomimetics10070459

**Published:** 2025-07-12

**Authors:** Kaichen Wang, Yan Shi, Junjie Chen, Yuchi Dai

**Affiliations:** 1 School of Mechanical and Electric Engineering, Changchun University of Science and Technology, Changchun 130022, China; 2 National Base of International Science and Technology Cooperation for Optics, Changchun 130022, China

**Keywords:** placoid scales, bionic microchannel heat sink, thermal management, numerical simulation

## Abstract

The rapid advancement of artificial intelligence continuously increases the demand for high computing power, leading to substantial rises in chip power consumption and heat generation. As a result, efficient thermal management has become essential. Inspired by the placoid scales on shark skin, we designed a bionic microchannel heat sink by introducing biomimetic structures on the inner channel surfaces to enhance heat dissipation. Numerical simulations are performed to investigate thermal behavior under different structural configurations. The results show that the arrangement, number, and inclination angle of the placoid structures significantly influence heat transfer by modifying flow patterns, enlarging the heat transfer area, and altering the thermal boundary layer. Notably, at a flow velocity of 2 m/s, the cooling performance differs significantly between inclination angles of 0° and 17°. Moreover, the influence of different quantities of placoid structures shows a consistent trend across various flow rates. These findings demonstrate that bionic surface structures can effectively improve the thermal performance of microchannel heat sinks, offering a promising strategy for high-performance chip cooling.

## 1. Introduction

In recent years, artificial intelligence (AI) has been widely recognized as a key driving force behind the new wave of the industrial revolution. However, the rapid growth in computational demands has led to a significant rise in power consumption. Meanwhile, the traditional form of Moore’s Law has gradually approached its physical limits, making the performance improvements of computing chips increasingly dependent on the integration of advanced fabrication processes and higher power input [1]. As power consumption continues to rise, heat accumulation in chips has become more severe, resulting in elevated overall temperatures and the formation of localized hot spots [2]. These thermal issues may lead to reduced computing speed, system crashes, or unexpected shutdowns, which not only compromise system stability but also accelerate hardware degradation [3]. Therefore, effective thermal management has become critical for improving operational reliability and extending the service life of electronic devices.

Among the various thermal management techniques, microchannel heat sinks have emerged as a promising solution for electronic cooling due to their compact structure and high heat transfer performance. Since the concept was first proposed by Tuckerman et al. in 1981 [4], microchannel heat sinks have been considered a viable approach to meet the increasing thermal dissipation demands of high-performance electronics [5]. Current research efforts have primarily focused on four aspects, including replacing the base material of microchannels [6,7,8], modifying the wettability of channel surfaces [9,10], optimizing coolant flow patterns [11,12], and innovating microchannel structural designs [13,14,15]. Among these, structural design has drawn particular interest in recent years, especially the integration of bionic features inspired by biological systems. The bio-inspired structure, by mimicking nature’s efficient forms, comprehensively outperforms conventional designs in heat dissipation performance, flow resistance, and temperature uniformity, making it the ideal choice for high-efficiency thermal management. Wang et al. [16] developed a biomimetic tree-shaped microchannel heat sink through optimized channel distribution and multi-level branching design, demonstrating a significant improvement in thermal dissipation performance. This work highlights the promising application of bio-inspired approaches in microchannel heat transfer technology. Such designs have demonstrated the ability to suppress turbulence generation within flow channels, reduce flow resistance and noise, and significantly enhance overall thermal performance [17].

To improve the flow and thermal performance of conventional smooth rectangular microchannels, researchers have introduced bio-inspired structures inspired by natural surfaces. For example, Wang et al. [18] designed a bio-inspired wall surface based on the microstructure of dragonfly wings and applied it to a rectangular channel. The bionic structure induced uniformly distributed high-speed vortices within the channel, enhancing fluid disturbance and weakening low-velocity, high-temperature regions near the wall, thereby significantly improving overall heat transfer performance. Fish scale structures also show strong potential in bio-inspired design. Dey et al. [19] applied fish scale geometry to the bottom of a microchannel and studied its flow and thermal behavior through both experiments and numerical simulations. The results demonstrated that the fish scale structure could induce an early transition from laminar to turbulent flow, enhancing the overall heat transfer rate. Building upon this, Goh et al. [20] incorporated fish scale-inspired inserts into an annular channel, achieving a convective heat transfer coefficient of up to 47.9 kW/m^2^·K under low-cost manufacturing conditions. They also established empirical correlations between the Nusselt number and friction factor, providing theoretical support for the design of efficient macro-scale heat exchangers. In microscale thermal management systems, simultaneously improving heat transfer performance, energy efficiency, and temperature uniformity remains a core research challenge [21]. Fu et al. [22] systematically reviewed marine drag reduction technologies and analyzed the influence of riblet count on the surface of shark placoid scales. They emphasized the importance of the “area factor” in shear stress calculations and experimentally confirmed the critical role of the riblet surface area in evaluating drag reduction performance. However, due to the complex microstructure of shark skin, current fabrication techniques still face challenges in achieving precise replication. Therefore, when designing shark skin-inspired surfaces, it is essential to simplify their morphological features and optimize them for manufacturability [23].

In summary, microchannel heat exchangers enable concentrated heat transfer within compact structures, while bio-inspired designs further enhance their thermal performance. Previous studies have thoroughly demonstrated the effectiveness of shark skin–inspired placoid scales in reducing fluid drag and noise [24,25]. Reduced drag typically leads to increased flow velocity, allowing for a larger volume of coolant to pass through the microchannels per unit time, thereby enhancing the overall heat dissipation performance. Thus, incorporating shark skin–inspired structures into microchannel heat sink design is supported by both theoretical and engineering perspectives. However, most existing studies have focused on single-scale morphology or isolated structural applications. The systematic optimization of the geometric parameters of shark scales and the coupled mechanisms between flow dynamics and heat transfer performance remain underexplored. Based on this background, the present study proposes a bio-inspired microchannel heat sink incorporating structural features derived from shark scales. A numerical simulation approach is employed to systematically investigate the effects of various geometric parameters—such as the number, arrangement, inclination angle of placoid scales, and riblet count—on fluid flow and heat transfer characteristics within the microchannel. The optimization of these parameters is further conducted with the aim of enhancing heat transfer performance and temperature uniformity. This study provides a novel design concept and theoretical support for the efficient thermal management of high-power computing chips.

## 2. Establishment and Validation of Physical Model

### 2.1. Model Description

**Figure 1:**
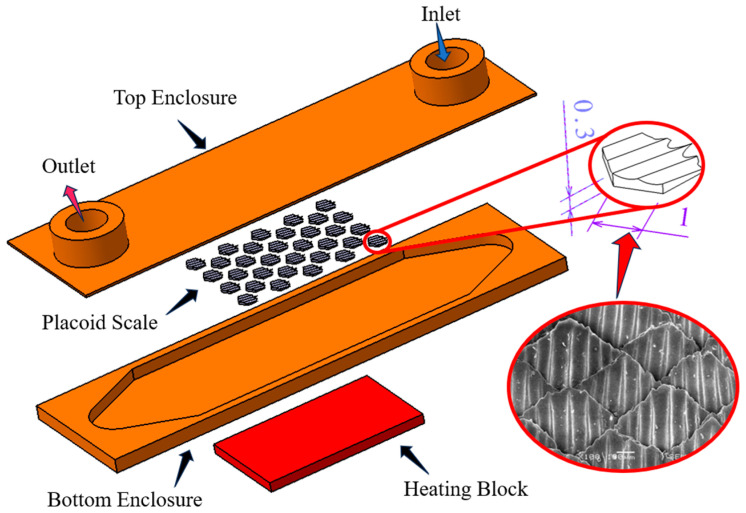
Demonstration of the bio-inspired microchannel heat sink model structure. Reproduced from Ref. [26] with permission from the *Journal of Materials Processing Technology*, copyright 2011.

The three-dimensional model of the shark skin-inspired microchannel heat sink is illustrated in Figure 1. It consisted of a fluid cavity serving as the flow channel, a series of bionic fin structures mimicking placoid scales on shark skin [26], and a base assembly including an inlet and outlet cap, as well as a heating block positioned at the bottom to simulate the heat source. The heating power was set based on the maximum turbo power (219 W) of commercially available Intel Core i9 processors. Deionized water at standard room temperature (298.15 K) was adopted as the coolant within the fluid domain [27]. The density is 997.0 kg/m^3^, the thermal conductivity is 0.606 W/(m·K), the kinematic viscosity is 8.93 × 10^−7^ m^2^/s, and the specific heat capacity is 4182 J/(kg·K). The fluid channel was designed with a height of 1.2 mm, a width of 12 mm, and a length of 20 mm, with an additional 5 mm development zone provided at both the inlet and outlet regions. The simulation employed copper as the material, which is widely used in thermal conductors such as heat pipes, and it is commonly utilized in the fabrication of heat sinks and radiator structures. The bionic fins were modeled based on actual electron microscopy images of placoid scales. Each fin featured a hexagonal base with a side length of 1 mm and a height of 0.3 mm.

### 2.2. Governing Equation Creation and Boundary Condition Setting

In this study, a three-dimensional numerical simulation of the shark skin-inspired microchannel heat sink was conducted using the ANSYS 2023 R2 Fluent platform [28]. The flow and heat transfer processes were solved using the incompressible steady-state Navier–Stokes equations. The boundary layer consists of three zones with a transition ratio of 0.272, a growth rate of 1.2, and a smooth transition offset method. The working fluid was assumed to be steady state, three-dimensional-incompressible, and with laminar flow and constant fluid properties. During the heat transfer process, thermal radiation and viscous dissipation were neglected, and a no-slip condition was applied at the fluid–solid interface. Under these assumptions, the governing equations for the fluid–solid coupled model was formulated as follows [29,30,31,32]:

Continuity equation:

(1) ∇⋅u=0

Momentum equations:

(2) $$\rho_{f}\left[\left(u\cdot \nabla \right)u\right]=-\nabla p+\mu_{f}\nabla^{2}u$$

Energy equation:

(3) $$\rho_{f}c_{pf}u\cdot \nabla T_{f}=k_{f}\nabla^{2}T_{f}\;\;\;\;\;\;\;\;\;\left(fluid\right)$$

(4) $$k_{s}\nabla^{2}T_{s}=0\;\;\;\;\;\;\;\;\;\;\left(solid\right)$$

Interface coupling condition:

(5) $$T_{f}=T_{s}$$

(6) $$k_{f}\frac{\partial T_{f}}{\partial n}=k_{s}\frac{\partial T_{S}}{\partial n}$$

The interaction between the energy equations of the fluid and solid regions is handled through interface boundary conditions, eliminating the need for additional source terms. At the interface, thermal equilibrium is enforced by setting the fluid temperature $T_{f}$ equal to the solid temperature $T_{S}$, as specified in Equation (5). Simultaneously, Equation (6) ensures that the heat flux remains continuous across the interface, thus satisfying energy conservation. In this context, $\rho_{f}$ represents the fluid density, u is the fluid velocity vector, p is the pressure, $\mu_{f}$ is the dynamic viscosity, $c_{p,f}$ denotes the specific heat capacity of the fluid, $k_{f}$ and $k_{s}$ are the thermal conductivities of the fluid and solid.

The accuracy of the numerical results was optimized by employing appropriate mesh structures and element quantities within a reasonable computational timeframe. A grid independence test was conducted to evaluate the precision of the pressure drop between the inlet and outlet, Δp, and the temperature difference between the inlet and outlet $T_{d}$ estimations across four distinct mesh densities. As presented in Table 1, the comparative results of Δp and $T_{d}$ for the model at $U_{ave}$= 8 m/s demonstrate that Mesh 3 exhibits minimal deviations of merely 0.09% in Δp and 0.19% in $T_{d}$ when compared with the finer Mesh 4. Consequently, Mesh 3 was selected, as it achieves an optimal balance between computational accuracy and time efficiency. Similar mesh schemes were subsequently established for other models in the study.

**Figure 2:**
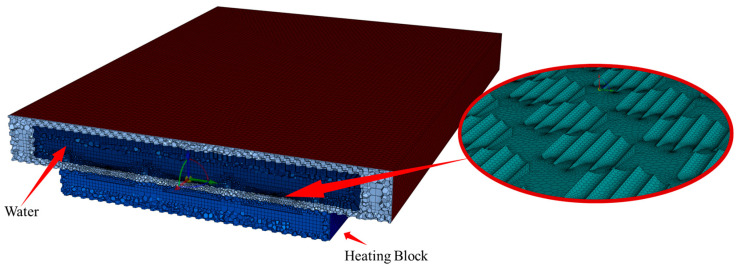
Bionic microchannel heat sink mesh model.

The numerical simulation model consisted of 4,897,380 nodes and 1,475,168 elements. The mesh configuration in the central region of the flow channel is shown in Figure 2. Due to the geometric complexity of the placoid scale structures, which included numerous sharp edges and curved surfaces, the poly-hexcore meshing strategy was employed. This technique generated structured hexahedral elements in the core region of the computational domain, thereby enhancing both numerical accuracy and computational efficiency. To accurately capture the fine-scale turbulence at the fluid–solid interface, the number of boundary layers was increased on the fluid side adjacent to the bio-inspired surface, further improving precision while maintaining acceptable computational cost.

### 2.3. Validation of Coupling Model

To validate the reliability of the simulation model, the Nusselt number and the Fanning friction factor were introduced as evaluation metrics. Since both parameters are derived from classical empirical correlations based on smooth-walled channels, an additional baseline model without bio-inspired structures was established for verification purposes.

The Nusselt number, a key dimensionless parameter in heat transfer analysis, was employed to evaluate the enhancement of convective heat transfer relative to pure conduction. It is widely used in thermal management systems such as coolers, heat exchangers, and microchannel heat sinks. The definition of the Nusselt number is given as follows:

(7) $$Nu=\frac{hD_{h}}{k}$$

where k is the thermal conductivity of water, h is the convective heat transfer coefficient, and $D_{h}$ is the hydraulic diameter. The relationship between h and $D_{h}$ is given by the following equation:

(8) $$h=\frac{q^{\prime \prime }}{\Delta T}$$

(9) $$D_{h}=\frac{4A_{C}}{2\left(b+w\right)}$$

(10) $$A_{C}=bw$$

where $q^{\prime \prime }$ is the heat flux, and ΔT is the temperature difference between the average wall temperature $T_{w,ave}$ and the average fluid temperature $T_{f,ave}$. b and w are the height and width of one mini-channel, and $A_{C}$ is the cross-sectional area of one channel [33].

Based on this, Gnielinski [34] and Dittus–Boelter [35] proposed classical empirical correlation formulas for calculating the Nusselt number, which were widely used to estimate the Nusselt number under turbulent forced convection conditions in tubes and subsequently evaluate heat transfer performance.

(11) $$C_{t}={\left(\frac{T_{f,ave}}{T_{w,ave}}\right)}^{-0.1}$$

where $C_{t}$ is the temperature correction factor.

(12) Nu=0.023Re0.8Pr0.4                    Dittus−Boelter

(13) Nu=f8Re−1000Pr1+12.7f8(Pr23−1)[1+DeL23]Ct                    Gnielinski

where Re is the Reynolds number and Pr is the Prandtl number, which is calculated as follows:

(14) Re=ρUaveDhμ

where μ is the dynamic viscosity of the fluid and $U_{ave}$ is the average inlet velocity.

The Fanning friction factor [36] was a dimensionless parameter used in fluid mechanics to describe the influence of wall shear resistance on fluid flow within pipes or channels. It was commonly employed in calculating pressure drops for internal flows, such as those in microchannels and conduits.

(15) $$f=-\frac{\left(\Delta p/L\right)D_{h}}{2\rho U_{ave}^{2}}$$

where L denotes the length of the flow channel.

Based on this foundation, Blasius [35] and Filonenko [32] proposed two classical empirical formulas for estimating the friction factor, a dimensionless parameter used to characterize flow resistance in fluid systems. These formulas were derived from experimental data and were applicable to pipes with varying flow conditions and surface smoothness.

(16) f=1.82lgRe−0.64−2                    Filonenko

(17) f=0.316Re−0.25                    Blasius

**Figure 3:**
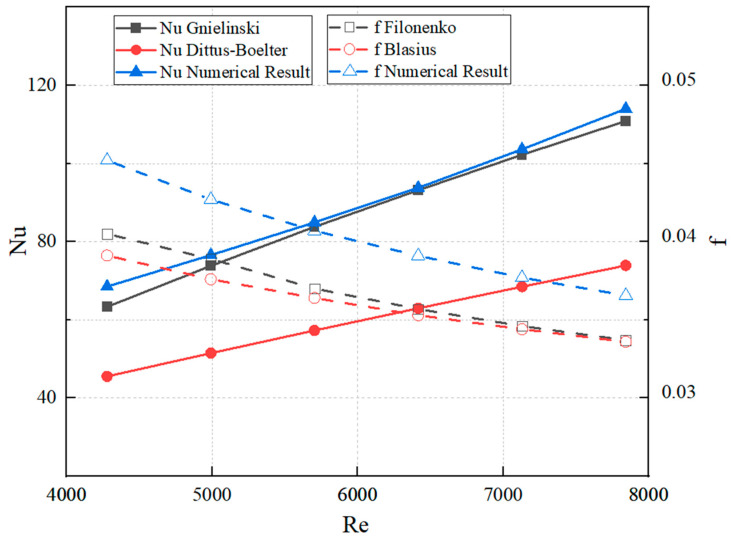
Comparison of Nusselt number and friction factor between numerical simulation results and empirical correlations.

The friction factor and Nusselt number obtained from numerical simulations in smooth channels were compared with the corresponding values calculated using empirical correlations. The comparison results are presented in Figure 3. The simulated values exhibited a clear consistency in trend with the theoretical predictions, indicating the reliability of the numerical simulation method.

## 3. Simulation and Validation of Bionic Microchannel Heat Sink

The effectiveness of the fluid model for the microchannel heat sink was validated by comparing the Nusselt number and friction factor. To enhance the cooling performance of the heat sink for heating block applications, a bionic design was introduced on the channel surfaces to improve heat transfer performance. The placoid scales on shark skin were known to influence the velocity gradient within the boundary layer, and the ridge-like ribs on their surfaces helped optimize the fluid structure and flow state in the turbulent boundary layer, thereby achieving a notable drag reduction effect [37]. Inspired by this biological prototype, a microchannel heat sink model with a bionic surface structure was designed.

### 3.1. Effect of Arrangement Pattern on Performance of MCHS

**Figure 4:**
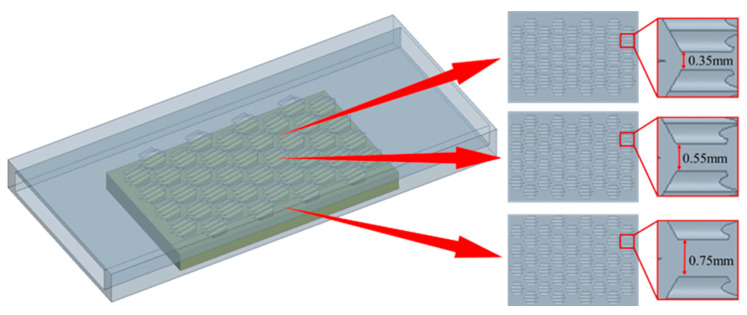
Different arrangement patterns of placoid scales.

The arrangement of placoid scales was found to guide fluid flow along specific directions, thereby stabilizing boundary layer development, reducing flow separation, and enhancing fluid adhesion. Accordingly, three different bionic flow channel designs were developed by varying the spacing between individual placoid scale structures, aiming to investigate the influence of the bionic scale arrangement on the performance of the heat exchanger. The specific configurations of the placoid scale arrangements are illustrated in Figure 4.

**Figure 5:**
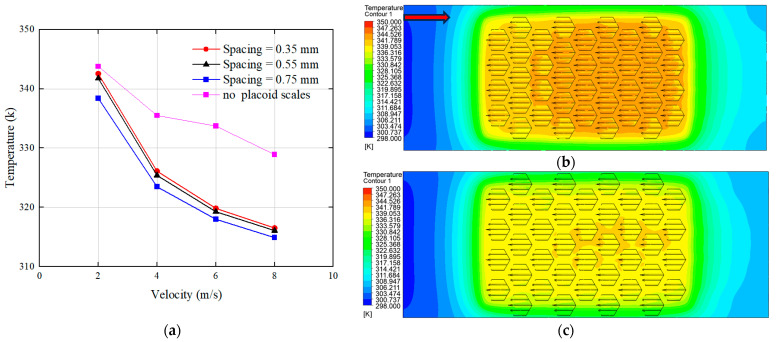
Effects of different structural spacings: (**a**) influence of structural spacing on the top surface temperature of the heating block; (**b**) temperature contour plot at a spacing of 0.35 mm; (**c**) temperature contour plot at a spacing of 0.75 mm. (The red arrow indicates the flow direction.)

At present, most commercially available liquid-cooled pump heads have a maximum flow velocity typically ranging from 6 to 10 m/s. For example, the AI-U8-V model from OCOCOO has a maximum flow velocity of 8 m/s. Therefore, the inlet flow velocity of the flow channel was set to 8 m/s. Considering that the CPU does not always operate at maximum power output, additional flow velocities of 6 m/s, 4 m/s, and 2 m/s were selected to represent cooling demands under medium- and low-heating block loads. Numerical simulations were then conducted to evaluate the thermal performance of the three bionic heat sink designs under different flow velocities, with a focus on temperature across the top of the heating block, as shown in Figure 5.

As shown in Figure 5a, the structure with the placoid scales exhibited significantly lower surface temperatures compared to the structure without placoid scales, and the temperature at the top surface of the heating block decreased with increasing spacing between the bionic structures. This effect was more pronounced at a flow velocity of 2 m/s compared to 8 m/s. The underlying cause of this phenomenon was further illustrated by the temperature contour plots of the fluid domain. Figure 5b,c presents the temperature distributions at spacing intervals of 0.35 mm and 0.75 mm, respectively, under a flow velocity of 2 m/s. It was observed that the temperature distribution in the fluid domain was more uniform at the larger spacing of 0.75 mm. In contrast, the temperature contour for the 0.35 mm spacing revealed distinct regions of elevated temperature within the gaps between the structures.

This phenomenon was caused by the narrow channel spacing of 0.35 mm, which restricted fluid flow between the structural gaps and led to the formation of turbulence within these regions. As a result, the local flow velocity significantly decreased, preventing the cooler fluid entering from the inlet from efficiently reaching the gaps to remove heat, thereby causing a rise in temperature. When the spacing was increased, the fluid was able to flow more freely between the structures, resulting in a more uniform temperature distribution across the entire fluid domain.

### 3.2. Effect of Different Number of Structures on Performance of MCHS

**Figure 6:**
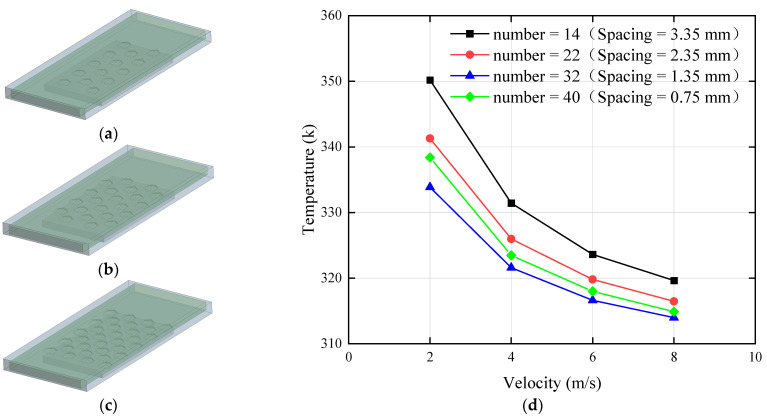
Effects of different structure quantities: (**a**) number of structures = 14; (**b**) number of structures = 22; (**c**) number of structures = 32; (**d**) influence of different structure quantities on the top surface temperature of the heating block.

By increasing the spacing between the placoid scale structures, the heat dissipation performance was improved. However, due to the limited surface area of the heat sink, further increases in spacing inevitably resulted in a reduction in the number of structures. Therefore, based on the initial design with 40 placoid scales (corresponding to a spacing of 0.75 mm), we further increased the structural spacing by reducing the number of scales while maintaining a uniform distribution across the bottom surface of the flow channel. The arrangements of 14 to 32 structures are illustrated in Figure 6a–c, and the thermal performance of the heat sinks with different numbers of placoid scales is shown in Figure 6d.

**Figure 7:**
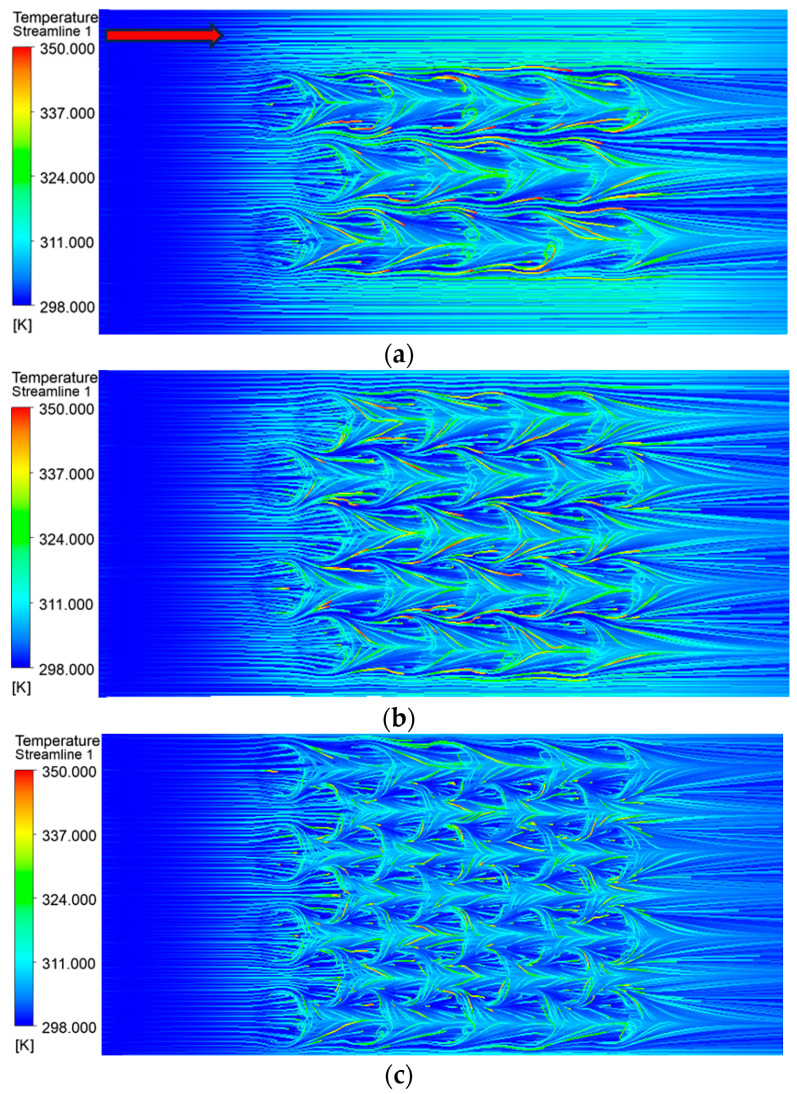
Temperature streamlines: (**a**) number of structures = 14; (**b**) number of structures = 22; (**c**) number of structures = 32. (The red arrow indicates the flow direction.)

By comparing the thermal performance of different quantities of placoid scale structures, it was found that the top surface temperature of the heating block reached its minimum when the structural spacing was 1.35 mm, corresponding to a total of 32 structures. When the spacing exceeded 1.35 mm, the temperature gradually increased with further increases in spacing. Conversely, when the spacing was reduced to 0.75 mm, the top surface temperature rose by approximately 2 °C. These results indicated that the heat sink achieved relatively optimal thermal performance when the structure quantity was 32 and the spacing reached 1.35 mm. To analyze the underlying cause of this phenomenon, streamlines were generated to investigate how the number of structures affected convective heat transfer within the fluid domain, as shown in Figure 7.

The temperature streamlines revealed that the fin structures on the placoid scales had a significant impact on the flow field. As the fluid passed over the fins, a noticeable temperature increase occurred, indicated in the figure by a shift from dark blue (lower temperature) to light blue (higher temperature). This demonstrated that the fin structures effectively transferred heat to the fluid, primarily due to the increased heat transfer surface area resulting from a greater number of fins per unit volume. When the number of bionic structures was 14, the total heat transfer area was 373.744 mm^2^, whereas increasing the number to 32 resulted in a heat transfer area of 391.414 mm^2^. The larger surface area allowed the fluid to absorb more heat conducted from the heating block, thereby improving heat exchange efficiency. Therefore, when designing the internal layout of the heat sink, it was essential to balance the number and distribution of the placoid scale structures. Excessively dense arrangements could hinder fluid flow within the channel, while an appropriate increase in the number of structures enhanced the contact area with the fluid, ensuring the overall thermal performance of the heat sink.

### 3.3. Effect of Inclination Angles on Performance of MCHS

**Figure 8:**
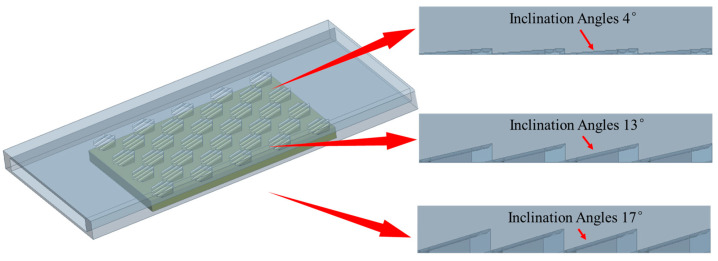
Different inclination angles of placoid scales.

By investigating the number and distribution of placoid scale structures, it was found that the configuration with 32 structures spaced at 1.35 mm exhibited superior thermal performance compared to the other designs. After determining the optimal quantity and distribution, further analysis was conducted on the influence of the fin inclination angle—a key geometric parameter—on the heat dissipation performance. Considering that the internal space of the heat sink should not be excessively large to avoid occupying too much chassis space, additional inclination angles of 0°, 4°, 13°, and 17° were introduced based on the original 8° design. The geometric configurations are shown in Figure 8.

**Figure 9:**
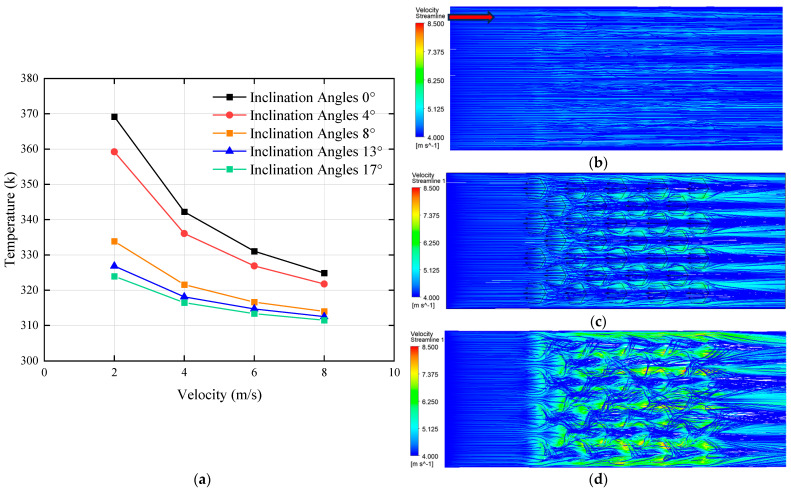
Effects of different inclination angles: (**a**) effect of various inclination angles on the temperature at the top surface of the heating block; (**b**) streamline velocity contours at an inclination angle of 4°; (**c**) streamline velocity contours at an inclination angle of 8°; (**d**) streamline velocity contours at an inclination angle of 17°. (The red arrow indicates the flow direction.)

By comparing the effects of inclination angles of 0°, 4°, 13°, and 17° on the top surface temperature of the heating block under different flow velocities, the relationship between fin inclination and temperature was established, as shown in Figure 9a. The results demonstrated that as the inclination angle increased, the temperature at the top of the heating block gradually decreased, although the rate of reduction became progressively smaller. To investigate the underlying reason why the inclination angle influenced the cooling effect, streamline velocity contour maps were extracted at a flow velocity of 4 m/s for inclination angles of 4°, 8°, and 17°, as shown in Figure 9b, Figure 9c and Figure 9d, respectively. The analysis revealed that increasing the inclination angle enhanced the flow velocity within the channel. This occurred because as the angle increased, the space above the bionic structures became more confined, forming a contraction from a larger to a smaller cross-section in the middle of the channel—resulting in a narrow throat followed by an expansion zone. This geometry resembled the Laval nozzle structure [38]. The emergence of temperature discontinuity within the 4–8° range confirms that an 8° angle provides adequate conditions for this phenomenon to occur. The streamline velocity contours clearly showed that the fluid experienced significant acceleration after passing through the structured region and then exited the channel at a higher velocity. However, due to the limited internal space of the flow channel, an excessive inclination angle reduced the top clearance and impeded the fluid’s passage. Pressure measurements at the inlet confirmed this effect. At a flow rate of 6 m/s, the inlet pressure was 38.08 kPa when the inclination angle was 8°, which increased to 63.48 kPa at 13° and further rose to 101.27 kPa at 17°. These results indicated that although increasing the inclination angle initially improved heat dissipation by accelerating the flow, the performance gain diminished progressively with larger angles. Effective heat transfer enhancement has been achieved at an 8° inclination. Beyond this angle, however, the performance gains diminish substantially as the resulting flow channel constriction creates a disproportionate increase in pressure drop without a corresponding enhancement in heat transfer performance. Meanwhile, the inlet pressure increased substantially, potentially placing excessive stress on the water pump. In practical applications, the pressure tolerance of the pump must be taken into account. Therefore, considering both performance and reliability, an inclination angle of 8° was selected as it achieves optimal balance—significantly enhancing heat transfer performance while maintaining pressure drop within acceptable limits.

### 3.4. Effect of Different Number of Fins on Performance of MCHS

**Figure 10:**
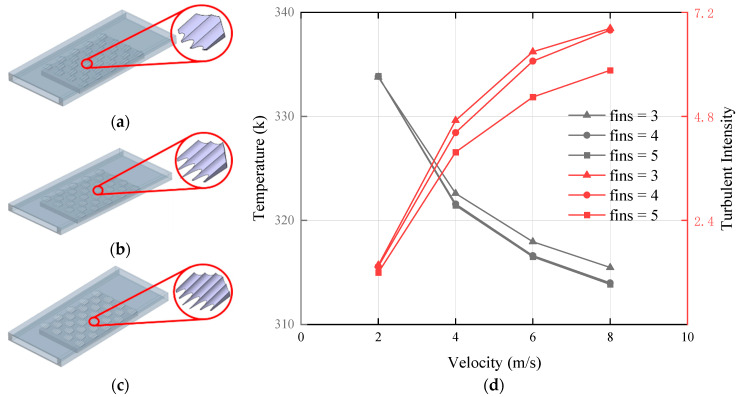
In the structure of 32 placoid scales, effects of different number of fins: (**a**) Number of fins = 3, (**b**) number of fins = 4, (**c**) number of fins = 5,(**d**) effect of different number of fins on the temperature at the top surface of the heating block and the turbulent intensity within the flow channel.

In addition to the inclination angle, the number of fins on the placoid scale also served as a critical parameter affecting fluid flow performance. Therefore, based on the original design with four fins and an inclination angle of 8°, we introduced modified structures with three and five fins, respectively, as shown in Figure 10a–c. The relationship between the number of fins and the temperature at the top surface of the heating block is illustrated in Figure 10d.

**Figure 11:**
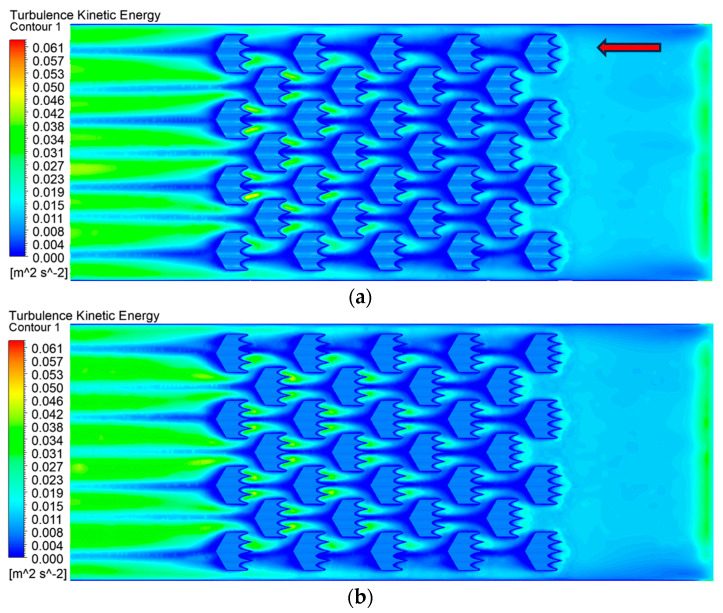

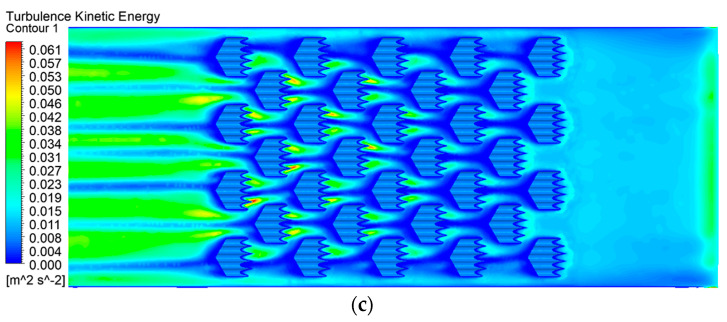
Effect of different fin numbers on turbulence kinetic energy: (**a**) number of fins = 3, (**b**) number of fins = 4, (**c**) number of fins = 5. (The red arrow indicates the flow direction.)

As shown by the analysis of the line chart, the heat dissipation performance was nearly identical when the number of fins was four or five, while a noticeable decline was observed when the number of fins was reduced to three. The magnitude of turbulent kinetic energy can serve as an indicator of flow resistance and objectively reflects the flow characteristics, which directly affect the thermal performance of heat sinks [39]. Therefore, we conducted a comparative analysis of turbulence intensity for these three configurations. It was found that the turbulent intensity reached its highest value with three fins and its lowest with five fins. This indicated that increasing the number of fins to five effectively suppressed turbulence, thereby accelerating the flow of fluid through the channel. To verify this assumption, the turbulence kinetic energy at the bottom of the flow field was simulated, as illustrated in Figure 11. When the fluid passed through the region covered by the bionic structure, the deepening blue color indicated the lowest-turbulence kinetic energy, confirming the suppression of turbulence over the fins. Moreover, Figure 10d showed that the turbulent intensity decreased with the increasing number of fins. These results demonstrated that the addition of an appropriate number of fins to the bionic structure not only increased the heat transfer area but also suppressed turbulent intensity, thereby enhancing fluid flow within the channel and further improving the thermal performance of the heat sink.

## 4. Conclusions

In this study, a novel biomimetic microchannel heat sink inspired by the placoid scale structures of shark skin was developed and numerically analyzed with the aim of enhancing thermal performance for high-power electronic chip cooling. The influence of key geometric parameters—including the arrangement, number, inclination angle of the biomimetic scales, and the number of fins—on thermal and hydrodynamic performance was systematically investigated.

The simulation results indicate that the incorporation of biomimetic structures significantly improves convective heat transfer by modulating local flow fields, increasing effective heat transfer surface area, and reshaping the thermal boundary layer. Among the tested configurations, an inter-scale spacing of 1.35 mm (corresponding to 32 structures) yielded the lowest temperature at the top of the heating block, indicating an optimal balance between flow resistance and heat exchange capability. Additionally, an inclination angle of 8° was identified as the most favorable, offering enhanced flow acceleration with an acceptable pressure drop, thereby ensuring operational compatibility with standard pump systems. Moreover, increasing the number of fins on the placoid structures was found to suppress turbulent intensity and reduce turbulence kinetic energy, which in turn contributed to more stable flow and improved overall thermal performance.

In conclusion, the proposed biomimetic design demonstrates significant potential for application in advanced microchannel cooling systems. The findings provide valuable theoretical and practical guidance for the structural optimization of heat sinks in high-performance electronic thermal management.

## Figures and Tables

**Table 1:** Results of grid independence tests with the model for Δp and $T_{d}$ at $U_{ave}$= 8 m/s.

No.	Grid Number	Δp (pa)	Deviation (%)	$T_{d}$ (K)	Deviation (%)
Mesh 1	590,067	27,797.30	2.79	17.961	3.29
Mesh 2	1,032,617	28,311.54	0.92	18.331	1.21
Mesh 3	1,475,168	28,568.66	0.09	18.516	0.19
Mesh 4	2,212,752	28,594.37	-	18.553	-

## Data Availability

The raw data supporting the conclusions of this article will be made available by the authors upon request.
